# Propofol attenuates odontogenic/osteogenic differentiation of human dental pulp stem cells in vitro

**DOI:** 10.1016/j.jds.2022.04.006

**Published:** 2022-04-20

**Authors:** Eun-Ji Choi, Cheul-Hong Kim, Ji-Young Yoon, Joo-Young Kim, Hyang-Sook Kim, Ji-Uk Yoon, Ah-Reum Cho, Eun-Jung Kim

**Affiliations:** a Department of Dental Anesthesia and Pain Medicine, School of Dentistry, Pusan National University, Dental Research Institute, Yangsan, Republic of Korea; b Research Institute for Convergence of Biomedical Science and Technology, Pusan National University Yangsan Hospital, Yangsan, Republic of Korea; c Department of Anesthesia and Pain Medicine, School of Medicine, Pusan National University, Yangsan, Republic of Korea; d Department of Anesthesia and Pain Medicine, School of Medicine, Pusan National University, Busan, Republic of Korea

**Keywords:** Bone tissue engineering, Dental pulp stem cell, Odontogenic/osteogenic differentiation, Human, Propofol

## Abstract

**Background/purpose:**

Various studies have used stem cells in the field of bone tissue engineering to repair bone defects. Dental pulp stem cells (DPSCs) have multipotent properties and can be acquired in a noninvasive manner; therefore, they are frequently used in experiments in regenerative medicine. The objective of this study was to investigate the odontogenic/osteogenic differentiation of human DPSCs (hDPSCs) using propofol, a widely used intravenous anesthetic agent.

**Materials and methods:**

Alkaline phosphatase (ALP) staining was used to investigate the effects of various concentrations of propofol (5, 20, 50 and 100 μM) on the osteogenic differentiation of hDPSCs. Real-time qPCR and Western blot analysis were used to detect the effect of propofol on the expression of odontogenic/osteogenic genes, such as DMP1, RUNX2, OCN, and BMP2. Odontogenic/osteogenic differentiation of hDPSCs was estimated at days 7 and 14.

**Results:**

ALP staining of hDPSCs was significantly decreased by propofol treatment. The mRNA expression of DMP1, RUNX2, OCN, and BMP2 decreased after propofol treatment for 14 days. The protein expression of DMP1 and BMP2 was decreased by propofol at days 7 and 14, and that of RUNX2 was decreased by propofol at day 14 only.

**Conclusion:**

Propofol attenuated odontogenic/osteogenic differentiation of hDPSCs in vitro. This result suggests that propofol, which is widely used for dental sedation, may inhibit the odontogenic/osteogenic differentiation of hDPSCs.

## Introduction

Bone disease and loss are recognized as important health problems because of the aging of the global population.^1^ In the field of dentistry, stem cell-based research for the repair of injured bone tissue has been pursued for a long time. Dental pulp stem cells (DPSCs) isolated from dental pulp tissue have mesenchymal stem cell (MSC) properties and can differentiate into multiple cell lineages, including adipogenic, odontogenic, osteogenic, chondrogenic, neurogenic, and myogenic, in vitro.^2^ The multi-lineage differentiation potential of DPSCs provides a source of cell-based therapies for regenerative medicine and tissue engineering.^3^ Furthermore, DPSCs are considered a promising source in stem cell-based regenerative medicine because of their simple isolation and culture, self-renewal capacity, high proliferation capacity, and ability to be cryopreserved.^4^, ^5^, ^6^

DPSCs are preferable and attractive tools for bone tissue regeneration and have been used in therapeutic stem cell applications.^7^^,^^8^ Osteogenic differentiation of DPSCs has been demonstrated in both in vitro and in vivo studies. The differentiation of DPSCs into functional osteoblasts is controlled by various cytokines and chemical stimuli, and DPSCs produce extracellular and mineralized matrices in vitro.^9^, ^10^, ^11^ The osteogenic differentiation potential of DPSCs has been demonstrated in animal models, and DPSCs seeded on collagen type I scaffold successfully repaired mandibular bone defects in human studies.^12^, ^13^, ^14^ Thus, various studies to demonstrate the osteogenic potential of DPSCs have persisted on account of the importance of bone tissue regeneration in the field of oral and maxillofacial defect therapeutics. However, further research is needed on the effects of various chemicals used in dental treatment on the osteogenic differentiation of DPSCs because the differentiation of functional osteoblasts can be affected by diverse chemicals.

Propofol is a short-acting intravenous anesthetic used for general anesthesia and moderate/deep sedation. Because propofol shows good sedation quality for invasive procedures, its use in dental sedation is gradually increasing.^15^^,^^16^ Several in vitro studies have investigated the effect of propofol on osteoblast differentiation. Lee et al. reported that propofol exerts beneficial effects on bone remodeling via the inhibition of osteoclastogenesis.^17^ Previous research has demonstrated that propofol increases bone nodular mineralization and the expression of bone-related proteins such as collagen type I, BMP2, osterix, and TGF-β1 in human osteoblasts under oxidative injury.^18^ However, the effect of propofol on the differentiation of DPSCs into odontoblasts/osteoblasts has not been evaluated.

In the present study, we investigated the effects of propofol on odontogenic/osteogenic differentiation of human DPSCs (hDPSCs) in vitro. We aimed to explore the pharmacological effect of propofol on the odontogenic/osteogenic differentiation of hDPSCs and to provide baseline evidence for in vivo studies and clinical applications in regenerative medicine.

## Materials and methods

### Culture of hDPSCs and propofol treatment

The hDPSCs were purchased from Lonza (PT-5025, Basel, Switzerland) and it is isolated from adult third molars collected during the extraction of a donor's ‘wisdom’ teeth. The hDPSCs were maintained in Eagle's minimum essential medium (MEM, Corning, Manassas, VA, USA), supplemented with 10% fetal bovine serum (Gibco BRL, Grand Island, NY, USA), 1% penicillin–streptomycin (Gibco BRL), and 5 μg/mL plasmocin (InvivoGen, San Diego, CA, USA) at 37 °C in a humidified incubator with 5% CO_2_, with a medium change twice a week. We used commercially available propofol (Fresenius Kabi Austria GmbH, Hafnerstrabe, Austria), which was dissolved into dimethyl sulfoxide (DMSO, Sigma–Aldrich, St Louis, MO, USA). The cultured cells were treated with propofol (5, 20, 50 and 100 μM).

### Assay of cell viability and proliferation

The hDPSCs were seeded into 24-well plates and treated with the indicated doses of propofol (5, 20, 50 and 100 μM) for 24, 48, or 72 h. At the end of the culture period, the cells were placed in a fresh medium containing 0.5 mg/mL 3-(4,5-dimethylthiazol)-2,5-diphenyl tetrazolium bromide (MTT, Thermo Fisher Scientific, Waltham, MA, USA) solution and incubated for 4 h before the addition of 200 μL DMSO. The resultant blue formazan products in hDPSCs were measured using a microplate reader at a wavelength of 570 nm.

### Osteogenic differentiation of DPSCs

For differentiation into the osteogenic lineage, hDPSCs at the third passage were cultured in osteogenic differentiation medium (ODM), α-MEM (Wellgene, Gyeongsan, Korea) containing 10 mM β-glycerophosphate, 50 μg/mL ascorbic acid, and 0.1 μM dexamethasone for 7 and 14 days in a humidified atmosphere of 5% CO_2_ at 37 °C and indicated dose of propofol was added to ODM. We used as vehicle control DMSO without propofol. The medium refreshed every 2–3 days.

### Alkaline phosphatase (ALP) staining and quantification

The hDPSCs were seeded in 48-well culture plates at a density of 5 × 10^4^ cells/well and osteogenic differentiation was induced using ODM with propofol, containing 10 mM β-glycerophosphate, 50 μg/mL ascorbic acid, and 0.1 μM dexamethasone for 7 and 14 days. To investigate the effect of propofol on the hDPSCs, various doses of propofol (0, 5, 20, 50 and 100 μM) were added to ODM and the medium refreshed every 2–3 days. For ALP staining, hDPSCs were rinsed with PBS containing 0.05% Tween-20 after aspiration of the medium, fixed with 4% paraformaldehyde for 2 min at room temperature, and stained using the StemTAG™ Alkaline Phosphatase Staining Kit (CBA-300, Cell Biolabs, San Diego, CA, USA) in accordance with the manufacturer's instructions. The stained cells were observed and photographed using phase-contrast microscopy. ALP staining was quantified by absorbance detection. Spectrophotometric absorbance of the samples was measured using a microplate reader (TECAN Infinite 200 PRO, Tecan Trading AG, Mannedorf, Switzerland).

### Real-time quantitative polymerase chain reaction (qPCR)

Total RNA was isolated using 500 μL of TRIzol reagent (Qiagen, Hilden, Germany), and 1 μg of mRNAs was reverse-transcribed with M-MLV (Promega, Madison, WI, USA), according to the manufacturer's instructions. Real-time PCR quantification was performed using SYBR Green premix (Qiagen). For real-time qPCR analysis, cDNAs were amplified with SYBR green PCR master mix (Qiagen) for 40 cycles, denaturation at 95 °C for 15 s, and amplification at 60 °C for 30 s in Rotor-GeneQ (Qiagen). Real-time qPCR data were analyzed using ABI 7500 Real-Time PCR system (ver.2.0.6, Applied Biosystems, Förster, CA, USA). The mRNA expression levels were normalized against glyceraldehyde-3-phosphatedehydrogenase (GAPDH). The primers used for PCR were as follows: ALP, 5′-GGACGCTGGGAAATCTGTG-3’ (forward) and 5′-CCATGATCACGTCAATGTCC-3’ (reverse); DMP1, 5′-AGGAAGTCTCGCATCT CAGAG-3’ (forward) and 5′-TGGAGTTGCTGTTTTCTGTAGAG-3’ (reverse); OCN, 5′-CAGCAAAGGTGCAGCCTTG-3’ (forward) and 5′-TGGGGCTCCCAGCCATTG-3’ (reverse); RUNX2, 5′-TCCCAGTATGAGAGTAGGTGTCC-3′ (forward) and 5′-GGCT CAGGTAGGAGGGGTAAGAC-3’ (reverse); and GAPDH, 5′-GGCGAGATCCCTCCAAAA TC-3’ (forward) and 5′-CAAATGAGCCCCAGCCTTC-3’ (reverse). All experiments were performed in triplicate.

### Western immunoblot analysis

Proteins were separated by SDS-PAGE and transferred to polyvinylidene difluoride membranes (Millipore, Billerica, MA, USA). Membranes were blocked with 4% skim milk in Tris-buffered saline containing 0.1% Tween 20 at room temperature. Proteins were detected with primary antibodies anti-DMP1 (Santa Cruz Biotechnology, Santa Cruz, CA, USA), anti-RUNX2 (MBL, Woburn, MA, USA), anti-BMP2 (Abcam, Cambridge, UK), anti-OCN (Abcam), and anti-β-actin (Santa Cruz Biotechnology), followed by incubation with horseradish peroxidase-conjugated secondary antibodies (Enzo Life Sciences, Minneapolis, MN, USA). It was detected using an enhanced chemiluminescence detection system (GE Healthcare, Buckinghamshire, UK), according to the manufacturer's instructions. Signals were detected using a Fusion Solo X (Vilber, Paris, France). The protein expression levels were normalized to that of β-actin. All Western blot analyses were repeated three times under the same conditions.

### Statistical analysis

Data represent the mean ± standard deviation obtained from at least three independent experiments. Statistical comparisons between groups were conducted using the student's t-test. Differences with *P* < 0.05 were regarded as significant and are denoted with an asterisk.

## Results

### Cell viability and proliferation assay of hDPSCs

**Figure 1:**
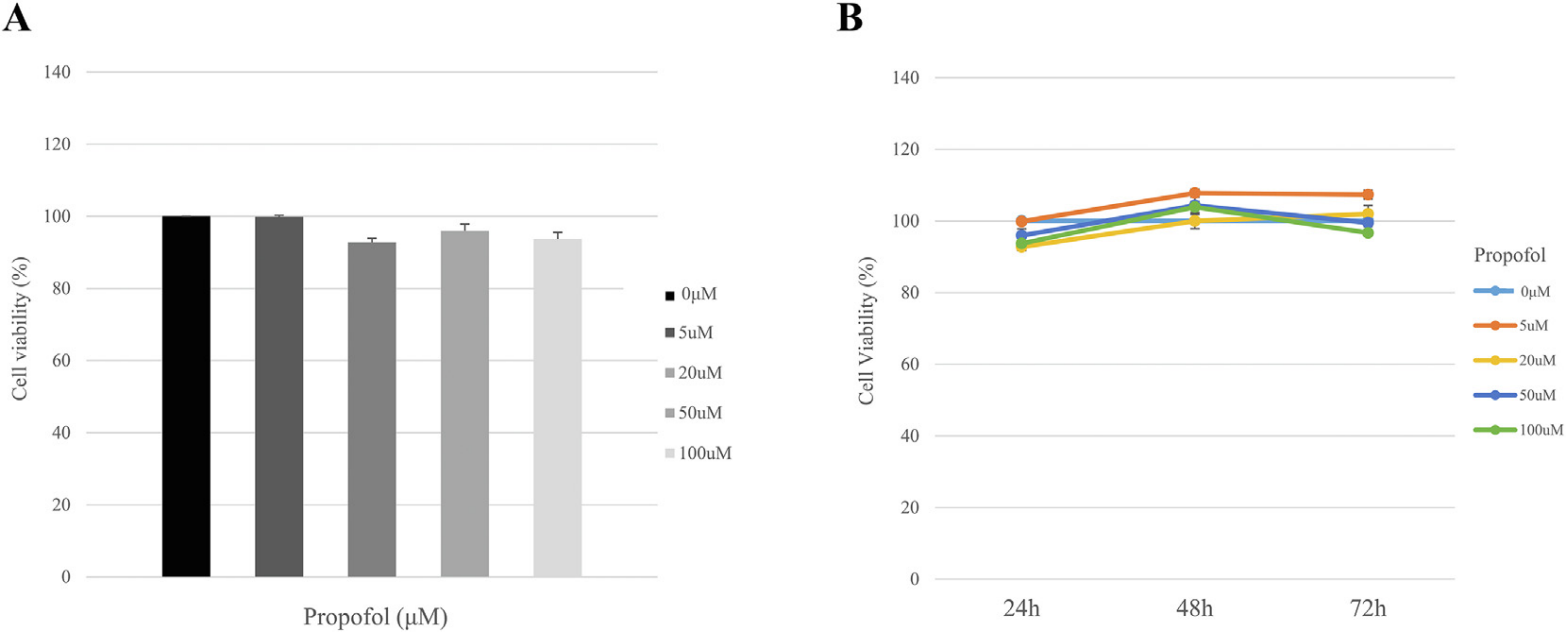
Effect of propofol on cell viability (A) and proliferation (B) of human dental pulp stem cells (hDPSCs) evaluated using MTT assay. The hDPSCs were treated with the indicated doses of propofol (0, 5, 20, 50, and 100 μM) for 24, 48, or 72 h.

To evaluate the effect of propofol on hDPSC viability, MTT assay was conducted. As shown in Fig. 1A, there was no significant difference in cell viability after propofol administration. In addition, there was no significant difference in cell viability after propofol treatment, even when observed up to 24, 48, and 72 h (Fig. 1B). Therefore, propofol has no cytotoxic effect and does not influence hDPSC proliferation.

### ALP staining and quantification

**Figure 2:**
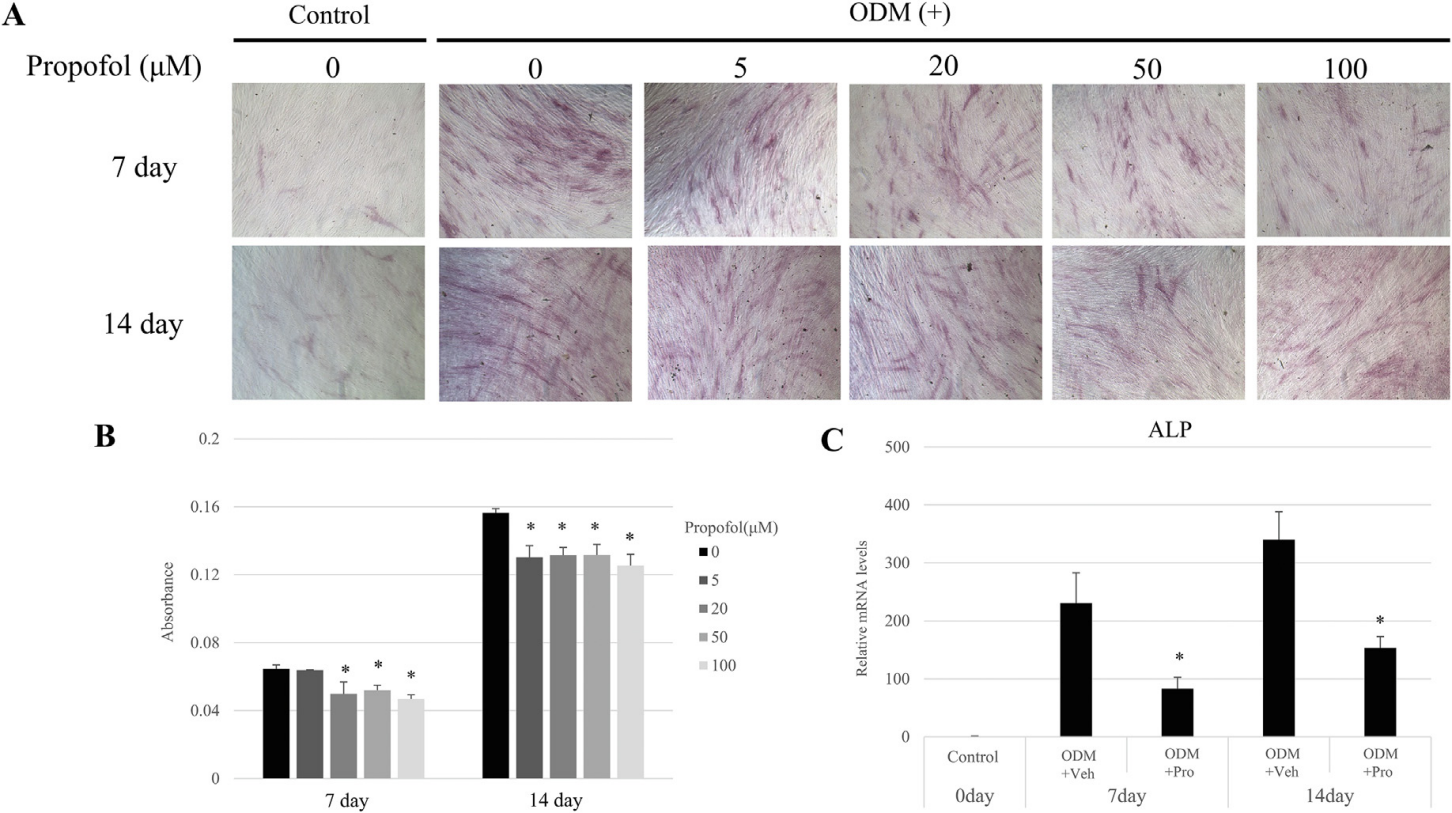
Effect of propofol on alkaline phosphatase (ALP) staining of human dental pulp stem cells (hDPSCs). (A) The hDPSCs were cultured in an osteogenic medium (ODM) with indicated concentrations of propofol for 7 and 14 days. ALP staining was carried out on 7 and 14 days. Stained cells were observed and photographed by a phase-contrast microscope. (B) Quantification of ALP staining was performed by absorbance detection using a microplate reader. ∗*P* < 0.05 compared to 0 μM propofol. (C) The mRNA expression of ALP after propofol treatment (50 μM) was analyzed by real-time qPCR at 7 and 14 days. ∗*P* < 0.05 compared to ODM + Veh. Pro: Propofol; Veh: Vehicle control.

The hDPSCs were cultured in ODM for 14 days, osteogenic differentiation was evaluated using ALP staining, and ALP-positive areas of hDPSCs were measured using a microplate reader on days 7 and 14. Additionally, ALP mRNA expression was estimated using real-time qPCR analysis. As shown in Fig. 2A and B, ALP staining and ALP-positive areas were decreased by propofol treatment (20, 50, and 100 μM) at 7 days and all concentrations (5, 20, 50, and 100 μM) compared with DPSCs cultured in ODM only at 14 days. However, no significant difference was observed in ALP absorbance between propofol concentrations. This result was accompanied by a decrease in ALP mRNA expression in propofol-treated hDPSCs (Fig. 2C). The mRNA expression of ALP decreased at 7 and 14 days after propofol treatment (50 μM).

### Real-time qPCR results for mRNA expression of odontogenic/osteogenic genes

**Figure 3:**
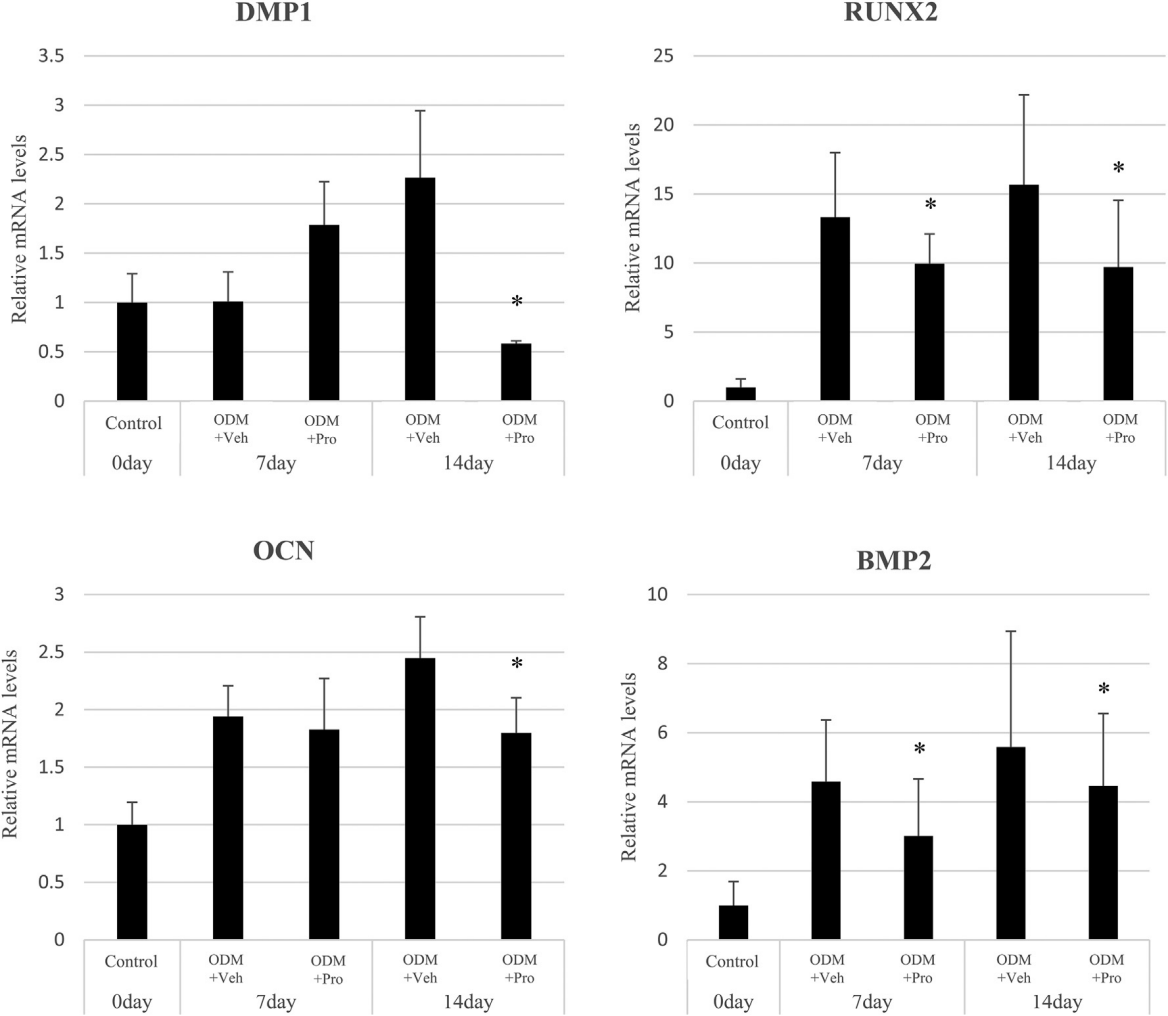
Effect of propofol on the mRNA expressions of odontogenic/osteogenic genes in human dental pulp stem cells (hDPSCs). The mRNA levels of DMP1, RUNX2, OCN, and BMP2 were evaluated using real-time qPCR in hDPSCs cultured in osteogenic media (ODM) with or without propofol (50 μM). The values were normalized against glyceraldehyde-3-phosphatedehydrogenase (GAPDH). All experiments were performed in triplicate. ∗*P* < 0.05 compared to ODM + Veh. Pro: Propofol; Veh: Vehicle control.

To investigate the influence of propofol (50 μM) on odontogenic/osteogenic differentiation of hDPSCs, mRNA expression of DMP1, odontoblastic differentiation markers RUNX2, OCN, and BMP2, and osteogenic differentiation markers were examined using real-time qPCR. The mRNA expression of the odontogenic marker DMP1 was significantly decreased in propofol-treated hDPSCs at 14 days but increased at 7 days compared to that in the untreated hDPSCs. As shown in Fig. 3, propofol treatment of hDPSCs significantly reduced the mRNA expression of the osteogenic markers RUNX2 and BMP2 at 7 and 14 days. OCN mRNA expression was significantly reduced by propofol treatment at 14 days.

### Western blot results for expression of odontogenic/osteogenic proteins

**Figure 4:**
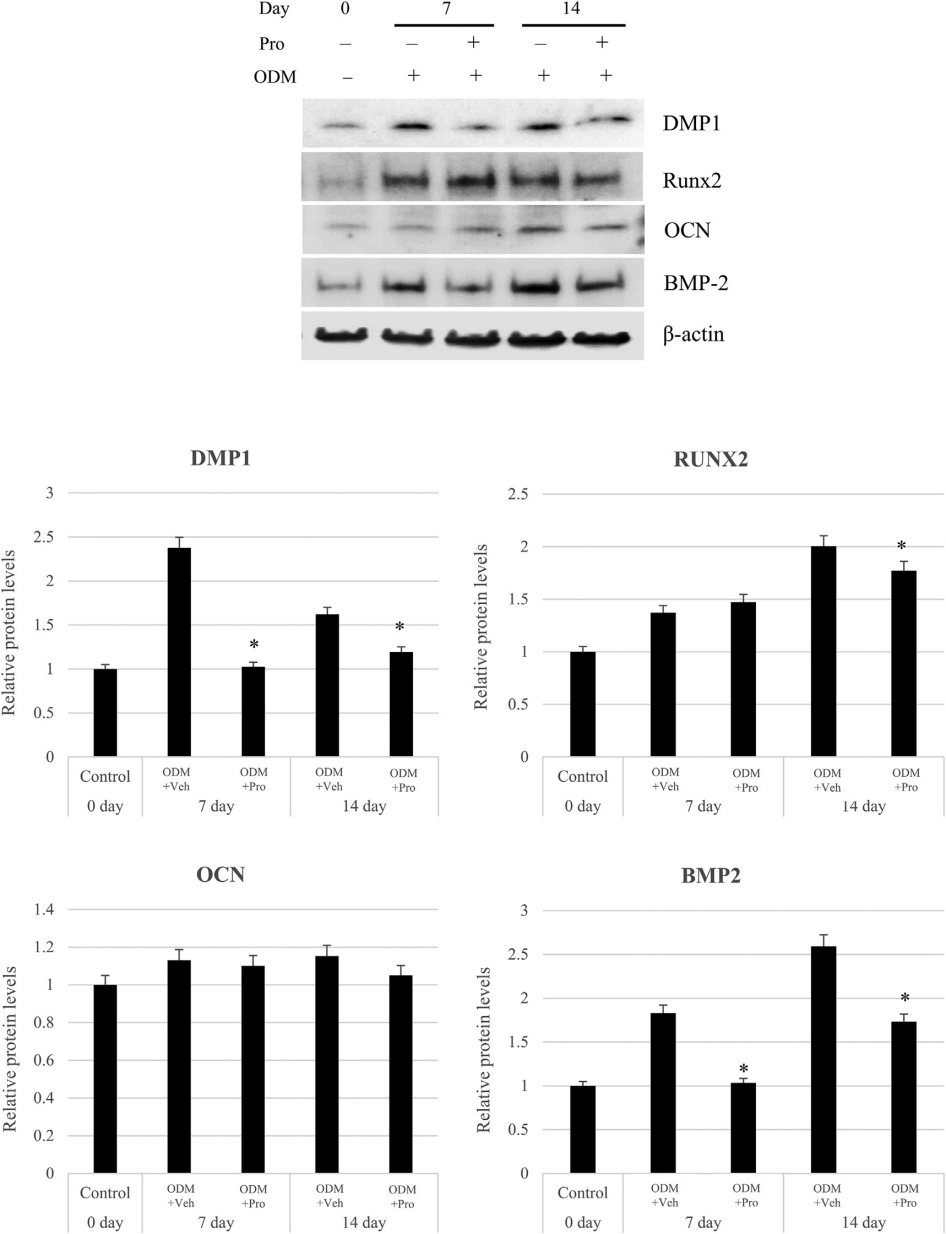
Effect of propofol on the protein expression of odontogenic/osteogenic genes in human dental pulp stem cells (hDPSCs). The protein expressions of DMP1, RUNX2, OCN, and BMP2 were examined using Western blot analysis in hDPSCs cultured in osteogenic media (ODM) with or without propofol (50 μM). Protein expression levels were normalized to that of β-actin. All Western blot analyses were repeated three times. ∗*P* < 0.05 compared to ODM + Veh. Pro: Propofol; Veh: Vehicle control.

The effect of propofol on protein expression of odontogenic/osteogenic genes was explored using Western blot analysis. It was performed with 50 μM propofol treatment. The protein expression of DMP1 was significantly reduced by propofol treatment after 7 and 14 days of differentiation. Among the osteogenic markers, the protein expression of BMP2 was significantly decreased by propofol treatment at 7 and 14 days, and RUNX2 was significantly decreased at 14 days. Although the protein expression of OCN was slightly decreased by propofol treatment, it was not significant (Fig. 4).

## Discussion

Bone marrow MSCs (BM-MSCs) have been used as a gold standard in bone tissue engineering to treat and repair injured bone tissue.^19^^,^^20^ However, there is a need to find other sources of MSCs to replace BM-MSCs because the process of BM-MSCs collection is painful and invasive. The craniofacial structure is a derivative of mesenchymal cells derived from the neural crest, and DPSCs are also derived from the neural crest.^21^^,^^22^ Therefore, DPSC is in the spotlight as stem cells that can replace BM-MSCs in bone tissue engineering. DPSCs and BM-MSCs have similar characteristics. All of them have the potential for multiple differentiation and a high proliferation capacity.^23^ In addition, DPSCs and BM-MSCs can be transplanted across barriers with a histocompatibility complex, without immune suppression.^24^ DPSCs are superior to BM-MSCs in some respects. DPSCs have many advantages, such as high efficiency in the process of extracting stem cells from pulp tissue, low morbidity due to anatomical damage caused by pulp collection, and anti-inflammatory properties.^25^^,^^26^ It has also been reported that the potential of DPSCs for proliferation into bone tissue and induction of mineralization is superior to that of BM-MSCs.^27^

In this study, propofol treatment decreased the protein and mRNA expression of DMP1, RUNX2, OCN, and BMP2. However, the reduction in mRNA expression by propofol treatment was clearer than the reduction in the protein expression of odontogenic/osteogenic genes. In addition, the inhibitory effect of propofol on the expression of odontogenic/osteogenic genes was more pronounced on day 14 than on day 7 of culture. RUNX2 is a transcription factor that is essential in the early stages of osteogenic differentiation of DPSCs.^28^ Real-time qPCR showed that mRNA expression of RUNX2 was significantly decreased by propofol treatment on days 7 and 14. However, a decrease in protein expression was observed only on day 14. The difference in mRNA and protein expression is expected to result from the time delay between transcriptional induction and protein level increase. It has been reported that induced transcription does not immediately lead to increased protein levels during state transition (e.g., cell differentiation) owing to maturation, export, and translation of mRNA taking some time.^29^ In addition, several previous studies have shown a synthesis delay between mRNA and protein.^30^, ^31^, ^32^

In this study, the results of real-time qPCR and western blotting revealed that the mRNA expression level of OCN decreased significantly at day 14 and at the same time, the protein level was slightly but not significantly decreased by propofol. OCN is a late-stage marker of mineralized bones.^33^^,^^34^ Therefore, if western blotting is performed after a longer incubation period, the protein level may significantly decrease. In another study, the osteogenic differentiation potential of DPSC was evaluated until day 28.^35^ Further research is required to investigate the effects of propofol on the expression of osteogenic genes.

Several studies have reported that propofol has a positive effect on osteoblastic osteogenesis.^17^^,^^18^ However, in our study, propofol decreased ALP staining and osteogenic gene expression in hDPSCs. This result is contrary to those of previous studies. In previous studies, the effect of propofol on osteogenic differentiation was examined using preosteoblasts,^17^^,^^18^ however, in this study, the effect of propofol on osteogenic differentiation was examined using stem cells such as DPSCs. Several studies have been conducted to determine how propofol affects the neural differentiation of stem cells. Liang et al. demonstrated that propofol inhibited neural stem cell proliferation, migration, and neuronal differentiation.^36^ Li et al. found that moderate and high concentrations of propofol interfered with the proliferation and differentiation of neural stem/progenitor cells.^37^ In a previous in vivo study, propofol enhanced the therapeutic effect of bone marrow mesenchymal stem cell transplantation for the recovery of the injured spinal cord in a rat model.^38^ This is the first study to investigate the effect of propofol on the odontogenic/osteogenic differentiation of mesenchymal stem cells.

It has been reported that the clinical blood concentrations of propofol were 0.8–1.0 μg/mL for awakening from anesthesia, 1–2 μg/mL for long-term intensive care unit sedation, and 3–11 μg/mL for maintaining the general anesthesia.^39^ In another study, they reported that propofol at 30 μM is close to clinical plasma concentrations.^40^ In this study, we used propofol at concentrations of 5, 20, 50 and 100 μM. These concentrations ranges about from 1 to 20 μg/mL. Therefore, the propofol concentrations used in this study are appropriate for clinical practice.

However, our study had some limitations. First, the decrease in the protein expression of odontogenic/osteogenic genes was not clear compared to the decrease in mRNA expression after propofol treatment. However, as mentioned earlier, there may be a time delay between mRNA and protein expression. In addition, it can be suggested that propofol has an inhibitory effect on the odontogenic/osteogenic differentiation of hDPSCs by reducing mRNA expression. Second, we used DPSCs derived from human for the experiment. There is a limitation in validity of the conclusion with the use of one source of DPSCs. Third, we did not perform experiments to reveal the signaling pathway related to the inhibitory effect of propofol on odontogenic/osteogenic differentiation of hDPSCs in this study. Further studies are required to investigate the signaling pathways involved in the results obtained in this study.

In conclusion, we showed that propofol attenuated the odontogenic/osteogenic differentiation of hDPSCs in vitro. This is the first in vitro study to evaluate the effect of propofol on hDPSC differentiation, especially regarding odontoblasts and osteoblast differentiation. Further studies are needed, nevertheless, this in vitro study is meaningful in that it provides evidence of propofol, which is widely used for dental sedation, may have an inhibitory effect on odontogenic/osteogenic differentiation of hDPSCs.

## Declaration of competing interest

The authors have no conflicts of interest relevant to this article.
